# Differences in the perceived role of the healthcare provider in delivering vascular health checks: a Q methodology study

**DOI:** 10.1186/1471-2296-14-172

**Published:** 2013-11-14

**Authors:** Stephanie Honey, Louise D Bryant, Jenny Murray, Kate Hill, Allan House

**Affiliations:** 1 Leeds Institute of Health Sciences, Charles Thackrah Building, University of Leeds, 101 Clarendon Road, Leeds LS2 9LJ, UK

**Keywords:** Healthcare professional attitudes, Lifestyle advice, Primary health care, Prevention, Cardiovascular diseases, Health behaviour, Nursing, General practitioner, Q methodology

## Abstract

**Background:**

The UK Department of Health introduced the National Health Service (NHS) Health Check Programme in April 2009 in an attempt to improve primary and secondary prevention of cardiovascular disease in the UK population and to reduce health inequalities. Healthcare professionals' attitudes towards giving lifestyle advice will influence how they interact with patients during consultations. We therefore sought to identify the attitudes of primary care healthcare professionals towards the delivery of lifestyle advice in the context of the NHS Health Check Programme.

**Methods:**

Fifty-two primary care healthcare professionals undertook a Q sort with 36 statements that represented a range of viewpoints about the importance of lifestyle change, medication, giving lifestyle advice in the primary care setting, and the individual, social and material factors that might impact on lifestyle related behaviour change. Sorts were analysed by-person using principal components analysis and varimax rotation.

**Results:**

Five statistically independent factors (accounts) reflected distinct views on the topic. Account 1 was supportive of initiatives like the NHS Health Check, and emphasised the importance of professionals working collaboratively with patients to facilitate lifestyle change. Account 2 expressed views on the potential overuse of statin medication and placed responsibility for lifestyle change with the patient. Account 3 viewed the healthcare professional role to be one of educator, emphasising the provision of information. Account 4 perceived lifestyle change to be difficult for patients and emphasised the need for healthcare professionals to be role models. Account 5 was inconsistent about the value of lifestyle change, or the role of healthcare professionals in promoting it, a finding that may be due to ambivalence about the health check or to lack of engagement with the Q sort task. We found no strong associations between any of the factors and, gender, role, age or ethnicity.

**Conclusions:**

Our findings suggest that healthcare professionals hold viewpoints that may influence how they interact with patients during health checks. When implementing programmes like the NHS Health Check, it would be useful to take healthcare professionals’ views into account. Attitudes and beliefs could be explored during training sessions, for example.

## Background

Cardiovascular disease is responsible for 40% of all deaths in the UK and impairs the quality of life of more than 4 million people [1]. In 2008 it was estimated that one fifth of hospital admissions were related to cardiovascular conditions. The UK Department of Health (DH) introduced the National Health Service (NHS) Health Check Programme in April 2009 as a vascular risk assessment service, available to everyone aged between 40 and 74 years [2]. The NHS is a healthcare system, publicly funded by taxes, that provides healthcare for all UK citizens based on their need for healthcare rather than their ability to pay for it. The Health Check Programme is carried out by general practitioners (GPs) and practice nurses within the primary health care system. Primary care provides the first point of contact for most patients and delivers treatment for common illnesses, screening services, management for long term conditions, such as coronary heart disease, and preventive services such as immunisations.

The aim of the Health Check is to estimate an individual’s risk of developing diseases affecting the cardiovascular system, including diabetes and kidney disease. The risk is calculated by means of a physical assessment including blood samples, questions about medical and family history and consideration of lifestyle risks. Patients considered to be at high risk will be given support with lifestyle modification and medication [3]. Modifiable risk factors for cardiovascular disease include overweight and obesity, an unhealthy diet, tobacco use, alcohol consumption and physical inactivity. Evidence suggests that if these factors are addressed, an individual’s risk could be reduced by over 80% [4].

The ability to achieve a healthy lifestyle may be affected by socio-economic status [5], a disadvantage that may be compounded by wider determinants of health such as poverty, poor housing, inadequate education and, possibly, reduced access to healthcare [6]. An important longer-term aim of the NHS Health Check Programme is to reduce inequalities in premature deaths from cardiovascular disease, although it is not made explicit how this aim is to be achieved.

There is evidence to suggest that professional attitudes interact with patient characteristics to influence how healthcare professionals deliver lifestyle advice [7][8][9][10]. For example Arber *et al.*[11] found that healthcare professionals were less likely to ask about smoking and alcohol consumption during screening for coronary heart disease, if the patient was female or more elderly. Some physicians may deliver less information to patients from lower socio-economic status, or minority ethnic, groups because they perceive them to be less educated, intelligent and rational [12]. In terms of the NHS health check itself, views on its effectiveness vary, as demonstrated by the range of on-line responses to a BMJ article querying the value of periodic health checks [13]. Some critics highlight evidence which questions the aims and value of the programme [14]. Studying one PCT, the NHS health checks failed to identify up to a third of people at high risk for type 2 diabetes [15].

In this study therefore, we sought to identify the range of views held by relevant primary care professionals towards giving lifestyle advice in the context of the NHS Health Check Programme. To do so we used Q methodology, a robust technique for identifying and examining distinct types of subjective opinion on social phenomena. A strength of choosing this approach is that Q methodology inherently deals with the ‘wholeness’ of a person’s view and allows for conflicting or ambivalent elements to be expressed; important when considering attitudes towards a relatively new social phenomenon where viewpoints, and reasoning behind those viewpoints, may be less stable. Q methodology has been used successfully to study related questions in health, such as the attitudes of healthcare providers towards health promotion [16], the views of diabetic patients on lifestyle choices [17] and attitudes towards healthy lifestyles in young people [18].

## Methods

### Q methodology overview

Q methodology combines quantitative research techniques and analysis with qualitative approaches to sampling and pattern interpretation [19]. It aims to detect the range of distinct points of view, on any given topic within a given population, by requiring participants to consider and respond to a set of predefined attitudinal statements on the topic under investigation [20]. To do this participants use a ranking technique (the Q sort) to express their view towards a diverse range of things already written or said about the topic [21]. The sorting patterns of the participants are analysed using by-person correlation and factor analytic techniques to identify distinct ‘clusters of like-mindedness’ [22], that is, the factors produced represent distinct patterns in the viewpoints of participants. Importantly, because Q methodology groups people according to their views, it helps identify where people with different personal characteristics, roles or experiences may share views. We use the word ‘factor’ in the methods section as this refers to the statistical output from the principal components analysis, but move on to using the term ‘account’ in the results and discussion sections to reflect the change from a statistical analysis to a qualitative interpretation of viewpoints.

### Creating the Q sample

This Q study was part of a wider programme of work ‘Improving the Prevention of Vascular Events in Primary Care’ (IMPROVE-PC), a National Institute for Health Research (NIHR) funded programme examining factors influencing take up of lifestyle advice in primary care. As part of IMPROVE-PC, systematic literature reviews and face-to-face interviews with healthcare professionals were conducted. We used the material collected during these earlier parts of the programme as the source for the Q sample, that is things ‘written or said’ about NHS health checks.

#### Literature review

The literature reviews had brought together primary research relating to the experiences of community-based healthcare providers responsible for cardiovascular prevention, and identified healthcare professional related factors that influence this delivery. Studies were included if they examined the views and attitudes of community-based healthcare professionals; were about the delivery of cardiovascular prevention and lifestyle advice; examined health behaviours (alcohol use, smoking, physical activity and diet); and related to relevant medical conditions, including obesity, cardiovascular disease, type 2 diabetes and, hypertension (Murray J, Honey S, Bara AC, Hill K, House AO: A qualitative synthesis reporting barriers and facilitators to delivery of lifestyle support in primary care to individuals at high risk of cardiovascular events, in preparation) (Bara AC, Murray J, Honey S, Hill K, House AO: A systematic review reporting the prevalence of factors that influence the delivery of lifestyle support in primary care to individuals at risk high of cardiovascular events, in preparation). We also drew on two literature reviews conducted within IMPROVE-PC that reported patient experience in this area [23,24].

#### Interviews

Transcripts from 49 interviews with primary care healthcare providers (including practice nurses, practice managers, healthcare assistants and GPs) were used as source material for the Q sample (Murray J, Fenton G, Hill K, Honey S, House AO: A qualitative study examining the attitudes and experiences of community based care providers delivering lifestyle support to patients at high risk of cardiovascular events, in preparation). Interviews had covered accounts of participants’ involvement with the NHS Health Checks, views on how the programme was working in primary care and the skills needed to deliver lifestyle advice and carry out the checks. The transcripts were scrutinised for material relevant to the aims of the project, and quotes were extracted from transcripts.

Statements collected from the literature and the interview sources represented a wide range of viewpoints about the NHS Health Check, giving lifestyle advice in the primary care setting and the individual, social and material factors that might impact on behaviour change. The statements, initially just over one hundred in total, were thematically analysed (JM) and organised under five categories: (1) NHS culture, (2) The care provider, (3) Patient barriers/facilitators, (4) Systemic effects, and (5) Past relationships with patients. Through a series of consultations, the research team (SH, LB, JM, KH, AH) revised and reduced the statements to a set of 36 items, and conducted a pilot Q-study with five primary healthcare professionals (SH, JM). The final Q-set of 36 items (see Table 1) was representative of the original, wider population of ‘things written or said’ about this topic.

**Table 1:** Factor arrays: scores against each factor by item

**Item**	**Statement**	**Factor 1**	**Factor 2**	**Factor 3**	**Factor 4**	**Factor 5**
1	Once someone’s on a statin, getting them to change their lifestyles is less important.	−2	−5	1	−4	−2
2	It’s better to put people on statins as healthy lifestyles don’t really reduce cardiovascular risk.	−2	−4	0	−4	0
3	Most people have heard it all before so lifestyle advice on its own isn’t very effective.	−1	1	−3	−2	3
4	Lifestyle change is too difficult and so it’s better to put people on statins.	−4	−4	−4	−3	−1
5	Health trainers and health educators can relate to people from deprived backgrounds in a way that healthcare providers can’t.	0	−1	−1	2	−2
6	Healthcare providers who smoke give smokers an easier time than those that don’t.	1	0	1	−3	1
7	In general people don’t know how to make lifestyle changes so it’s better if healthcare providers tell them what to do.	−2	- 3	2	4	2
8	Patients should be given the opportunity to lower their cholesterol/BP by lifestyle change before being put on medication.	1	5	4	2	0
9	Listening to people’s problems and helping them to resolve their problems, is a worthwhile part of lifestyle change support.	4	3	1	3	1
10	When a practice can offer an in-house weight management clinic, there’s little point referring patients elsewhere.	1	0	−1	0	−4
11	It’s unfair that people from more deprived areas are given more resources to help them change their lifestyles.	−3	−2	0	−1	−1
12	Suggesting ways to overcome barriers to lifestyle change is more productive than working with patients to get them to identify their own solutions.	0	−1	3	0	1
13	Patients don’t tend to listen to what healthcare providers say about lifestyle change.	0	−3	0	−2	1
14	The GP/nurse can only do so much because takeaways and supermarkets have a much bigger influence on peoples eating habits.	2	1	1	−2	3
15	For people who don’t want to change their lifestyle, it’s better to say ‘my door is always open’ than spend ages discussing their problems.	3	3	0	0	2
16	People should take personal responsibility and sort their own lifestyles out instead of expecting others to do it for them.	1	2	−2	−2	−3
17	Barriers to lifestyle change are mainly just excuses.	0	0	3	−1	−3
18	It costs too much to eat a healthy diet.	−1	−2	−1	0	−1
19	It’s up to the patient to tell me if there are any specific reasons why they can’t try to change their lifestyles.	1	−1	−2	1	−4
20	Identifying and working through peoples’ personal barriers to lifestyle change can make a difference to whether or not they actually try to change.	5	4	−1	2	1
21	People who don’t know about healthy lifestyles probably aren’t interested anyway.	−3	−2	−4	0	0
22	Checking on peoples’ emotional status should be a routine part of a cardiovascular health check.	3	−1	1	1	5
23	There’s no point discussing lifestyle change with people who are stressed or depressed.	−1	0	−3	−1	0
24	Patients are more likely to take notice of lifestyle advice if the healthcare provider sets a good example themselves.	2	2	2	5	−1
25	People are too busy to cook healthy meals or exercise.	0	0	−2	3	2
26	Getting a partner on side is an important part of supporting people to change their lifestyle.	3	4	4	2	2
27	It’s pointless living a healthy lifestyle because we are all going to die of something anyway.	−4	2	−3	−1	−1
28	If patients don’t understand what they’ve been told about lifestyle change and cardiovascular risk they will ask the healthcare professional to explain it.	−1	0	−1	1	4
29	Educated people find it easier to change their lifestyles.	2	2	5	−3	0
30	There’s no point going over stuff if people don’t want to change.	−1	3	2	−5	−3
31	If you know a patient well you know how much or little information they need.	2	1	3	3	0
32	Some people are beyond help.	−5	1	0	0	−2
33	Barriers to lifestyle change are hard to overcome so it’s best to stick to standard healthy lifestyle advice.	0	−3	0	−1	4
34	It’s better to go softly with the truth (e.g. play down the risk of cardiovascular disease) so that people aren’t put off coming back.	−2	−1	−2	1	−5
35	Because very few people are successful at making lifestyle change, discussing it in any depth isn’t worth it.	−3	−2	−5	1	−2
36	Understanding the impact of social factors leads to better discussions on lifestyle change.	4	1	2	4	3

### Ethical approval

Ethical approval for the study was obtained from the Newcastle and North Tyneside 1 National Research Ethics Committee (10.H0906/52).

### Participants

In Q methodology participants are recruited on the basis of their ability to express informed views on the research topic, rather than their statistical representativeness [18]. For this study we used purposive sampling to recruit from the range of healthcare professionals involved in the delivery of the NHS Health Check or subsequent lifestyle advice. We also recruited from areas with a range of deprivation scores, including many from less advantaged areas. The participants (n = 52) comprised GP principals (n = 10), GP registrars (n = 11), primary care nurses (n = 17), healthcare assistants (n = 13), pharmacists based in primary care (n = 1). Participants were recruited from two primary care trusts (as they were then known) in the North of England between November 2011 and March 2012.

### Procedure: administering the Q sort

**Figure 1:**
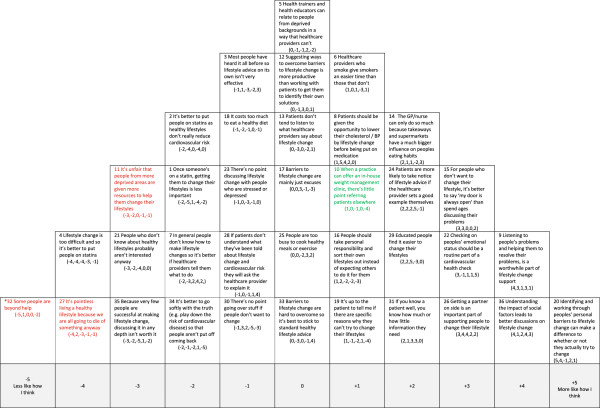
**Account 1: Healthcare professional as active facilitator of lifestyle change. Red text** = Item ranked LOWER than in other Account (* significantly so at *p < 0.01*); **Green text** = Items ranked HIGHER than in other Account (* significantly so at *p < 0.01*).

Each healthcare professional was visited individually by a researcher at their place of work, given written study information and asked to give written consent to participate. Verbal instructions about how to complete the Q sorting were given. The Q-set was shuffled prior to administration and participants were asked to read the statements and, in a series of steps, to rank the items by placing the cards in a quasi-normal distribution on the Q-grid (see, for example, Figure 1). Each column of the grid represented a response from +5 (more like how I think) to −5 (less like how I think). In this way, a set of ranked data was collected for each participant, the pattern of the Q sort representing the relationship of the items to their own perspective about the health checks and the importance of primary care services in improving patient lifestyle. The participants were asked to reflect on their Q sort patterns and to move statements around until they were happy with their arrangement. The final grid comprised the participant’s Q sort, which was recorded on a data collection sheet. The sorting task took participants 20 minutes to complete on average.

### Statistical analysis and interpretation

A total of 52 Q sorts were included in the analysis. The Q sort data were managed and analysed using a dedicated Q methodology software package PQMethod, V. 2.1 [25]. Q methodology uses data reduction techniques, mainly factor analysis or principal component analysis (PCA) to identify relationships between individual Q sorts; in this study each factor (or principal component) represents a highly inter-correlated cluster of Q sorts, that is, the items were sorted in a statistically similar way. Each cluster can be considered to represent a distinct point of view on the given topic.

PCA assigns an eigenvalue to each of the factors, which indicates the size of the factor, and is the amount of variance in the correlation matrix for which it accounts. Eight of the unrotated factors produced by PQMethod had eigenvalues greater than one, which means they each contributed to the total explained variance at a level greater than one single variable (in Q method this equates to one participant). The percentages of explained variance for these eight factors were calculated as 41%, 7%, 5%, 5%, 4%, 4%, 3% and 3% respectively.

We used strategies described by Watts and Stenner to identify the maximum number of interpretable and distinct viewpoints to take forward from these eight, for example, considering only those factors with at least two Q sorts loading at 0.43 (*p* <0.01) on one factor only [19]. By considering each factor in light of the comments participants made during sorting and the qualitative interviews conducted with other health professionals (see ‘Interviews’ section), we concluded that a five-factor solution was the ‘best fit’ for the data. Each of the factors had at least three Q sorts loading highly and significantly (*p* < 0.01) on one factor only. These highly loading Q sorts are known as exemplar sorts in that they best exemplify the viewpoint represented by the factor. A weighted averaging formula [20] was then applied to the exemplar sorts to create a composite Q sort which can then be said to represent each factor (see Table 1). The composite Q sorts were reconstructed on sorting grids to aid interpretation, for example, Figure 1 represents the composite Q sort of the Factor 1 exemplars.

During interpretation, particular attention was given to the statements placed at the extreme ends of the grid, that is, closer to the ‘more like how I think’ or ‘less like how I think’ anchors, and the statements identified as statistically distinguishing for each factor at *p* < 0.01 and *p* <0.05. We also considered the statements that were ranked higher or lower in this factor when compared to other factors.

**Figure 2:**
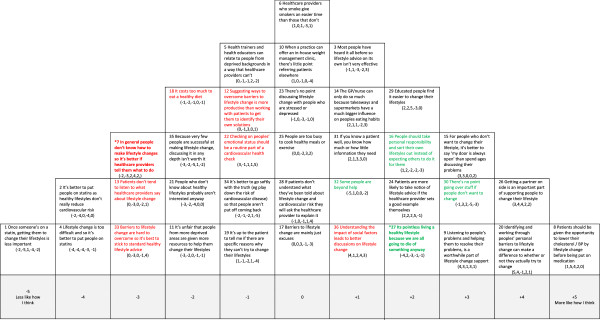
**Account 2: Lifestyle change not medication: but patients responsible for change. Red text** = Item ranked LOWER than in other Account (* significantly so at *p < 0.01*); **Green text** = Items ranked HIGHER than in other Account (* significantly so at *p < 0.01*).

**Figure 3:**
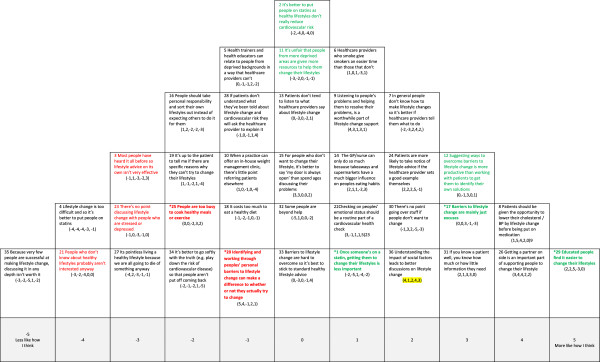
**Account 3: Healthcare professional as educator. Red text** = Item ranked LOWER than in other Account (* significantly so at *p < 0.01*); **Green text** = Items ranked HIGHER than in other Account (* significantly so at *p < 0.01*).

**Figure 4:**
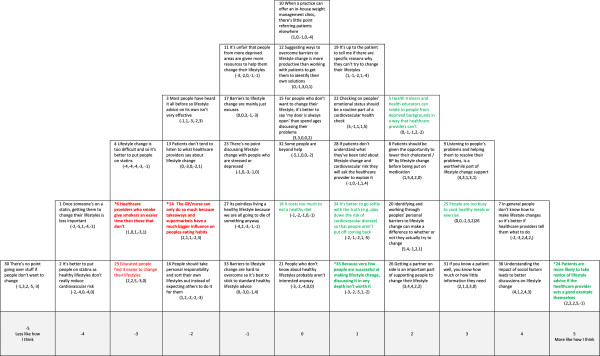
**Account 4: Change can be difficult: healthcare professional as role model with influence. Red text** = Item ranked LOWER than in other Account (* significantly so at *p < 0.01*); **Green text** = Items ranked HIGHER than in other Account (* significantly so at *p < 0.01*).

**Figure 5:**
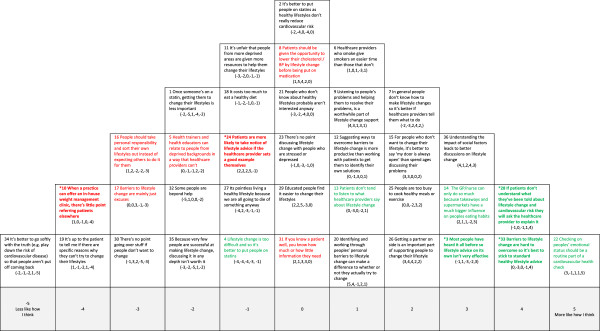
**The limited role of healthcare professionals in lifestyle change. Red text** = Item ranked LOWER than in other Account (* significantly so at *p < 0.01*); **Green text** = Items ranked HIGHER than in other Account (* significantly so at *p < 0.01*).

In Figures 1,2,3,4 and 5 the red text indicates items ranked lower than in other accounts. The green text indicates items ranked higher than in other accounts.

## Results

### Factor accounts

Table 2 provides the list of participants whose Q sorts exemplified each factor and the percentage variance that each factor accounted for. The interpreted factor arrays are, from now on, referred to as ‘accounts’ to reflect the change from a statistical analysis to a qualitative interpretation. The accounts represent the views, attitudes and perceptions of groups of healthcare professionals towards giving lifestyle advice in the context of the NHS Health Check Programme

**Table 2:** Characteristics of exemplar participants by Account

**Account**	**Descriptive label**	**% of total variance accounted for**	**Characteristics of exemplar participants**
1	Healthcare professional as active facilitator of lifestyle change	21	Female Health Care Assistant, age 33, White British
			Female Health Care Assistant, age 49, White British
			Female Health Care Assistant, age 65, White British
			Female Practice Nurse, age 54, White British
			Female Practice Nurse, age 55, White British
			Female Practice Nurse, age 35, White British
			Female General Practitioner, age 39, South Asian
			Female General Practitioner, age 42, Mixed
			Male General Practitioner, age 42, White British
			Male General Practitioner, age 44, White British
			Male General Practitioner, age 31, White British
2	Lifestyle change not medication: but patients responsible for change	14	Female Community Pharmacist, age 56, White British
			Female Health Care Assistant, age 45, White British
			Female Practice Nurse, age 59, White British
			Female Practice Nurse, age 52, White British
			Female General Practitioner Registrar, age 29, White British
			Female General Practitioner Registrar, age 31, White British
			Female General Practitioner, age 54, White British
			Male General Practitioner, age 50, White British
			Male General Practitioner Registrar, age 40, South Asian
			Male General Practitioner Registrar, age 30, South Asian
3	Healthcare professional as educator	8	Female Practice Nurse, age 55, White British
			Female Practice Nurse, age 37, White British
			Female General Practitioner Registrar, age 30, Mixed
4	Change can be difficult: healthcare professional as role model with influence	13	Female Practice Nurse, age 44, Black
			Female Practice Nurse, age 49, South Asian
			Male General Practitioner Registrar, age 36, Black
5	The limited role of healthcare professionals in lifestyle change	6	Female Health Care Assistant, age 61, White British
			Female Health Care Assistant, age 45, White British
			Male General Practitioner Registrar, age 28, White British

#### Account 1: healthcare professional as active facilitator of lifestyle change

Central to this account is the belief that patients can change their lifestyle and that it is the role of healthcare professionals to facilitate this change. This account is supportive of current lifestyle change policies and of an active, collaborative role for health professionals in primary care interventions such as the NHS Health Check (see Figure 1).

There is a strong belief in the ability of healthcare professionals to motivate patients to change their lifestyle. This viewpoint favours a shared, discursive approach to facilitating lifestyle change: working through barriers, listening, having an ‘open door policy’ for those unwilling or unable to change now (#20/+5, #9/+4, #15/+3 versus #7/-2). There was a clear rejection of pessimism about the possibility of lifestyle change, (#32/-5, #4/-4, #21/-3, #23/-1, #35/-3). There was also rejection of the fatalistic belief that as ‘people will die anyway’ lifestyle change is pointless (#27/-4).

This account recognises the importance of being aware of the social circumstances that influence people’s ability to improve their lifestyle, so that discussions about change are informed by an individual’s circumstances (#36/+4). There is a rejection of the idea that increasing resources for those in deprived circumstances is unfair (#11/-3) suggesting an awareness of health inequalities as linked to material resources and the role of the food industry (#14/+2). It is acknowledged that those with higher levels of education, for example, may find lifestyle change easier (#29/+2), and that those who may find the ideas more difficult may not always make this known to healthcare professionals (#28/-1).

Attitudes towards the use of medication (statins) were not the strongest theme in this account, but there was a view that as lifestyle change is possible, opting for medication as a first line treatment is not the best strategy (#4/-4, #2/-2, #1/-2, #8/+1).

#### Account 2: lifestyle change not medication: but patients responsible for change

In this account the most strongly expressed views are those in relation to the use of medication (see Figure 2). There is a clear view that people should be given the opportunity to improve their lifestyle before being put on statins (#8/+5, #1/-5, #2/-4, #4/-4). If a healthy lifestyle can be achieved this is the preferred treatment option (#8/+5).

Account 2 places more emphasis on the patient’s personal responsibility for change than Account 1. While professional input to support a healthy lifestyle is, like Account 1, considered important (#20/+4, #9/+3, #13/-3), patients have to be personally motivated to change (#16/+2). It is the role of healthcare professionals to help patients identify their personal barriers to change (#20/+4), and patients who are motivated and willing to change can benefit from lifestyle advice. It is not, however, the role of healthcare professionals to tell people what to do (#7/-3, #12/-1). This account does not consider understanding of social or material factors as particularly important in lifestyle change discussions (#36/+1, #18/-2), although it is recognised that patients with a higher level of education may find it easier to change their lifestyles (#29/+2).

Unlike Account 1, there is agreement that some people cannot be helped (#32/+1) or do not want to listen to advice about lifestyle change (#30/+3). For those people, therefore, the healthcare professional’s responsibility is to ‘keep their door open’ (#15/+3). There is also a view that the value of lifestyle advice is limited as people have ‘heard it all before’ (#3/+1). There is also a, possibly fatalistic, belief that ‘as everyone will die of something’, the focus on healthy lifestyles may be pointless to some degree (#27/+2). Agreement with this item significantly discriminated this view from all other accounts, which seems contradictory to the healthcare professionals’ belief in lifestyle change, but may be associated with the view that healthcare professionals are limited in what they can achieve. The patient is ultimately responsible for their choices and lifestyle.

#### Account 3: healthcare professional as educator

This account places the healthcare professional in a more traditional and directive role as an educator and emphasises the importance of communicating knowledge as the route to improving lifestyles (see Figure 3). In contrast to Account 1, where the healthcare provider is regarded as a facilitator, here the healthcare professional is regarded as an expert. There is the strongest agreement in this account that patients with higher levels of education find it easier to make lifestyle changes (#29/+5), but a view that information and lifestyle advice is valuable to everyone (#3/-3, #23/-3). Healthcare professionals, as experts, can judge how much or how little information different patients need (#31/+3). There was strong disagreement with the assumption that those who do not know about the importance of a healthy lifestyle will be uninterested in change (#21/-4). As some people don’t know how to make lifestyle changes, or won’t ask, the onus is on healthcare professionals to provide direction (#12/+3, #7/+2, #28/-1).

This account places significantly less importance than Accounts 1 and 2 on helping people to identify their own barriers (#20/-1) and there is a view, also setting this account apart from others, that perceived barriers to change were mainly ‘excuses’ (#17/+3). For example, there was significant disagreement that people are ‘too busy’ to eat healthily or exercise, or that lifestyle change is too difficult (#25/-2, #4/-4). Similarly, social and material factors were not seen as real impediments to lifestyle change (#11/0, #18/-1, #14/+1), although there was recognition that understanding social factors could improve discussions with the patient (#36/+2). As in Accounts 1 and 2 there is a view that lifestyle change should be attempted before medication is introduced (#8/+4, #4/-4), but once a patient is on medication lifestyle change may become less important (#1/+1).

#### Account 4: change can be difficult: healthcare professional as role model with influence

In this account, the healthcare professional is seen as the main influence on patient lifestyle change (see Figure 4). However, in contrast to the role of collaborator (Account 1) or educator (Account 3) this account believes healthcare professionals should ‘lead by example’, and be healthy lifestyle role models for their patients (#24/+5). There is clear value in healthcare professionals telling people how to change their lifestyle (#7/+4) and disagreement that patients don’t listen to what they say (#13/-2). Healthcare professionals can judge how to personalise information at an appropriate level for their patients (#31/+3). This account is distinguished from all others by a belief that healthcare professionals have an important influence on lifestyle, despite operating within a ‘fast food’ culture (#14/-2).

This account emphasises working with the patient’s personal social circumstances (#36/+4) and taking account of personal barriers and difficulties (#9/+3). It is recognised that for some people, living a healthy lifestyle may be difficult due to time or money pressures (#18/0, #25/+3): these are valid barriers rather than just excuses. Unlike any other account this view did not accept that people with higher levels of education necessarily find changing their lifestyle easier (#29/-3). Although there is a recognition that, as success rates can be low (#35/+1), healthcare professionals should persevere with patients who appear unwilling to change (#30/-5). In common with accounts 1, 2 and 3 lifestyle change was considered the first line of approach rather than medication (#1/-4, #3/-2, #4/-3, #8/+2).

#### Account 5: the limited role of healthcare professionals in lifestyle change

This account appeared to contain some negativity towards the provision of lifestyle advice.

The ability of healthcare providers to change patient lifestyle in light of other personal, societal and cultural factors is questioned (see Figure 5). There is a belief that, in reality, the influence of healthcare professionals on lifestyle change is limited (#24/-1). Healthcare professionals have to compete with cultural forces such as takeaways and supermarkets that sell and promote unhealthy food (#14/+3), and barriers such as the lack of time to cook healthy meals and to exercise (#25/+2). These healthcare professionals have no strong feelings about medications (#2/0, #8/0) and may believe that, while lifestyle change should be given a chance, all strategies should be considered when trying to lower cardiovascular risk.

On the other hand, healthcare professionals should not play down the risks associated with an unhealthy lifestyle (#34/-5). They need to give healthy lifestyle advice but this may not be very effective on its own (#3/+3) and there is an acknowledgement that lifestyle change can be difficult (#33/+3). Individuals do not bear all responsibility for the lifestyle they lead (#16/-3). Personal and social barriers are real (#17/-3) and should be discussed with the patient (#36/+3). There is also a belief that emotional status is very important to health and not just lifestyle (#22/+5).

The account appears internally inconsistent in parts about the value of lifestyle change or the role of healthcare professionals in promoting it. Such inconsistency may be due to ambivalence about the health check or a lack of engagement with the Q sort task.

### Consensus items

Two items identified consensus, or lack of significant difference, across the accounts. Item 11 (‘It is unfair that people from more deprived backgrounds are given more resources to help them change their lifestyles’) was ranked between −3 and 0, with no account agreeing that providing additional health resources for the poorest in society was ‘unfair’. The second consensus item, number 18 (‘It costs too much to eat a healthy diet’), was ranked from −2 to 0, suggesting that all accounts felt eating healthily could be achieved within a limited budget. These items had been included to help identify views on material deprivation as a cause of social inequalities in health. They both failed to discriminate significantly between accounts, however, and reasons for this are considered in the Discussion.

### Factor membership

The characteristics of exemplar participants for each account are shown in Table 2. Both men and women were represented in all factors, with the exception of Account 3, the health professional as educator, where all three exemplars were female. However, no conclusions can be made about whether this viewpoint is gendered or not due to the qualitative nature of the study. Representatives from all the main professional groups are included across the five accounts although a greater number of GPs exemplified the views represented in Account 2 than 1.

### Themes across accounts

During the interpretation phase we were not only interested in identifying patterns of attitudes within each account, but also between the different accounts. Factors incorporated different views within a number of clear themes, which help identify where viewpoints are similar or different in relation to understanding how health checks and lifestyle advice may be delivered in practice. The three themes are now presented.

#### Responsibility for lifestyle change: individual versus healthcare professional

Views differed across accounts in terms of who was responsible for patient lifestyle change. Accounts 1, 3 and 4 viewed healthcare professionals as having a very significant role in influencing change. Account 1 emphasised an active partnership approach with a shared responsibility between professional and patient, whereas Accounts 3 and 4 emphasised the responsibility of the healthcare professional as an educator or as a role model, with patients having less responsibility in terms of being active partners in the change process. Account 2 emphasised the patient’s responsibility for their own lifestyle and health, with the role of healthcare professionals being limited in terms of making change happen. Account 5 was inconsistent about how much influence health professionals could actually have on patient lifestyle.

#### Consultation style: collaborative approach versus didactic

The different accounts highlighted a range of preferred consultation styles associated with lifestyle advice in the participants. Account 1 was the strongest advocate of the collaborative approach to lifestyle change, very much in line with ‘shared decision making’ and patient centred healthcare models. Account 2 appeared to prefer a more ‘hands off’ approach: if patients did not take responsibility for lifestyle change, the health professional could do little. Accounts 3 and 4 favoured a more traditional ‘health education’ approach to advice and information, but still tailored to the individual patient. Account 5 emphasised a patient-centred approach, but not necessarily with an emphasis on lifestyle change.

#### The role of statin medication versus lifestyle change

Statins are a common pharmacological tool in the control of blood cholesterol levels. Across accounts different perspectives on the role of statins in relation to lifestyle change were apparent. Account 2 expressed strongest views about the benefits of lifestyle change over medication and, in line with the view about personal responsibility for health, the importance of behaviour change as a first line of treatment. Account 5 held a somewhat opposing view in that the value of medication was seen, especially for patients who for a variety of emotional, social and material factors find lifestyle change difficult. The other three accounts held broadly similar views: lifestyle change first, medication second with some variation on the role of lifestyle change once statins were being taken.

## Discussion

Analysis of the Q sorts identified five accounts which represent the viewpoints of groups of primary care healthcare professionals. Account 1 saw healthcare professionals as pro-active facilitators of lifestyle change. Account 2 considered that patients should take responsibility for their behaviour, emphasising the importance of lifestyle change as opposed to medication. Account 3 emphasised the educational role of healthcare professionals. Account 4 recognised that lifestyle change can be difficult and thought that healthcare professionals could be role models. Account 5 felt that the health professional’s role in supporting lifestyle change was limited due to multiple external influences, and there was less confidence in the value of this advice.

### Communication styles

Understanding of healthcare professionals’ attitudes to the NHS Health Checks, and the delivery of related advice, is an important step in understanding different ways in which lifestyle-related consultations may be conducted. Beliefs about the value of lifestyle advice and health checks might affect the consultation in ways that affect certain groups of people differentially. Each of the five accounts suggests a different approach to the consultation, the implications of which have been identified in the literature.

The socio-economic status of patients can affect doctor-patient communication and patients from lower socio-economic status groups may receive less information and advice [26]. Conversely, patients from higher socio-economic status groups may receive more information [27]. In 1991, Street [28] reported that physicians gave more information to better educated patients and suggested that many physicians assumed that less educated patients did not understand the information given to them. Compounding this, Kaplan *et al.*[29] found that patients with lower educational achievement participated less in consultations with physicians. More recently, Fiscella *et al.* found that family physician communication style was more directive during consultations with less educated patients [30]: the physicians spent less time answering questions or discussing health issues with these patients and more time on physical examination. This may particularly affect the promotion of behaviour change, as such discussions can be complex [31]. Taira [32] considered how patients’ incomes were related to unhealthy behaviours and the likelihood that healthcare professionals would discuss socio-economic issues during consultations. The study found that doctors were less likely to discuss diet and exercise, but more likely to discuss smoking cessation, with patients in lower socio-economic status groups.

Some aspects of our accounts supported these other research findings. The view that people with lower levels of educational achievement may find it harder to use health information to change their lifestyles was seen in views across most of the accounts but is particularly evident in Account 3. The views expressed in Accounts 3 and 4, where the healthcare professional is seen as an educator and information giver, also suggest that healthcare professionals assume they can make judgements about the amount of information appropriate to their patients. However, Johansson *et al.* showed that GPs often misperceive the needs of their patients, as regards lifestyle change information, and do not provide enough in-depth counselling [33,34]. Exemplars of Account 1 may be more likely to encourage all patients to participate in lifestyle consultations, although as we did not observe consultations in this study this is a supposition only.

### Health inequalities

The literature suggests that health professionals’ attitudes to the NHS Health Checks might have an effect on health inequalities, however our results did not demonstrate very clear findings in terms of view differentiation by social or material factors. This is in part due to the limited number of items in the Q set directly related to the role of social (items 14, 25, 29) or material factors (items 11 and 18). We found the development of these statements problematic as we did not want to ask about health inequalities too directly for fear of encouraging ‘socially desirable’ responses [35]. Possibly, as a result of this concern, we diluted the ability to assess attitudes towards social inequalities and the health check in this study. As identified previously, two of the items (11 and 8) were designed to identify views on material causes of health inequalities but did not discriminate between accounts. One explanation is that the health professionals in this study did not view material factors as very important considerations in lifestyle change. Alternatively, the items may have been worded in a way that made them ambiguous or hard to disagree with. Item 11 required participants to express whether it is ‘unfair’ to increase lifestyle change resources to those living in deprived areas. Participants may have found it difficult to suggest that people living in poverty did not deserve more resources; alternatively they may not have agreed that people in such circumstances actually did receive more resources. In relation to Item 8, eating healthily may be affordable at a purely cost per food item level, but at the same time it is known that people on low incomes may not have the skills or other resources required to support a balanced diet [36].

However, there were indicators that some participants held views on how social inequalities are reflected in health inequality. Account 4 expressed a view that changing to healthy diet and exercise habits require resources not at the disposal of less affluent people. Account 5 recognised the wider social forces associated with a ‘fast food culture’ and their implications for lifestyle change advocated by healthcare professionals, but this was not clearly aligned with a strong view on, for example, higher costs associated with a ‘healthy diet’. Account 4 most clearly indicated that understanding the social context of a patient helped with discussions about lifestyle change, but this appeared to be related to the immediate social context, such as time to cook and a supportive partner, rather than wider societal level disadvantage. In addition, accounts 1, 2 and 3 agreed that patients with a higher level of education found lifestyle change easier. Education is a common proxy for social class and it is recognised that people in higher socio-economic status groups have more positive attitudes towards, and greater levels of ‘healthy behaviours’ than those in lower socio-economic status groups [6]. Account 4, which rejected the idea that patients with higher levels of education found lifestyle change easier, were the group most likely to emphasise the difficulty of lifestyle change and the importance of health professional intervention.

### Implications

It is possible to see how the healthcare professionals’ attitudes we identified could influence the delivery of lifestyle advice in primary care. A collaborative approach, as exemplified by Account 1, may be most helpful for those where financial and social barriers may be present. A style that emphasises the responsibility of the individual, as exemplified by Account 2, may work best for people with higher levels of social and financial resources, but may disadvantage those who need more active support. An approach that emphasises the educational role of the healthcare provider (Account 3) may be beneficial to patients who prefer a more directive style, but health education, in terms of advice or information alone has not been shown to be effective in motivating lifestyle change [37]. A style which uses the healthcare provider as a role model, (Account 4), may be useful if the healthcare professional could describe how they made changes, including any barriers they overcame in order to reach their goals. On the other hand, this ‘advice’ may be discounted in those who believe they do not have the same available resources as those advising them.

These implications are postulated only, as we did not observe consultations directly nor assess their outcomes. We do not suggest that any one viewpoint should be taken as a role model; as identified earlier, evidence is lacking on the effectiveness of the health checks. More work is required to assess how or whether different attitudes to the delivery of health checks and lifestyle advice influence the effectiveness of such programmes. It has been noted elsewhere that the attitudes of nurses and doctors differ in the delivery of cardiovascular health promotion. One study, for example, found that more practice nurses than GPs felt it was their duty to carry out health promotion. In addition significantly more practice nurses felt they could offer effective lifestyle counselling [38]. It is important to note that, in our study, the factors did not show any strong associations with gender, age, role or ethnicity, although a greater number of GPs exemplified the views represented in Account 2 than Account 1.

### Limitations of the study

Q methodology studies may include interviews with the participants, to gain more insight into the placing of items at the extreme ends of the grid, for example. We were not able to do this due to time constraints during data collection as the study was carried out during the influenza vaccination season, a very busy time for healthcare professionals working in primary care. We did not therefore have further exploratory information to help support the interpretation of the factors and were not always able to explain the placing of some items. For example, the view that Account 4 did not think health professionals who smoked would be more lenient on patients who smoked (Item 6), despite the emphasis this factor placed on the healthcare provider as role model, appeared somewhat paradoxical. Account 5 was internally inconsistent and interpretation may have been aided by qualitative data to reveal more about the responses given. Nevertheless, we felt it was important to keep and interpret Account 5 because we recognized similar inconsistent or ambivalent attitudes during the qualitative interviews conducted as part of a separate strand of the research programme (Murray J, Fenton G, Hill K, Honey S, House AO: A qualitative study examining the attitudes and experiences of community based care providers delivering lifestyle support to patients at high risk of cardiovascular events, in preparation). This interview study examined the views of community based healthcare providers delivering lifestyle support to patients at high risk of cardiovascular events. These interviewees seemed to know what they *should* do but questioned the ‘point’ of doing it. Some participants in the Q study may therefore have had difficulty sorting the statements in relation to what they did in practice, as regards giving lifestyle advice, as opposed to what they thought they should be doing. In addition some participants put a lot of thought into the exercise, whereas others appeared to complete the exercise very quickly and, possibly, gave less considered responses.

## Conclusion

We sought to examine the attitudes of healthcare professionals to the delivery of lifestyle advice in the NHS Health Check Programme. Previous evidence suggests that support for patient lifestyle change could help to reduce health inequalities [5] and that the attitudes of healthcare professionals can influence the way in which that support is given [26]. While our findings also suggest that healthcare professionals have views that are likely to influence how they interact with patients, we cannot draw firm conclusions on how they contribute to health inequalities. It would be beneficial, therefore, to conduct observational studies alongside a Q study to investigate interactions between patients and healthcare professionals to further explore the impact of different viewpoints identified here on the actual delivery of lifestyle advice.

## Competing interests

There are no competing interests.

## Authors’ contributions

SH made substantial contributions to the design of the study, acquisition of data, analysis and interpretation of data, drafting the manuscript and revising it critically for important intellectual content. LB made substantial contributions to the analysis and interpretation of data, drafting the manuscript and revising it critically for important intellectual content. JM; KH made substantial contributions to the design of the study, analysis and interpretation of data, and revising the manuscript critically for important intellectual content. AH made substantial contributions to the conception and design of the study and revising the manuscript critically for important intellectual content. All authors have given final approval of the version to be published.

## Authors’ information

Stephanie Honey, RN, RM, BSc, MSc, PhD, Research Fellow.

Louise Bryant, PhD, CPsychol, Associate Professor in Medical Psychology.

Jenni Murray, BSc, MSc, PhD, Senior Research Fellow.

Kate Hill, BSc, MSc, PhD, Senior Research Fellow.

Allan House BSc. MBBS, MRCP, MRCPsych DM, Professor of Liaison Psychiatry.

## Pre-publication history

The pre-publication history for this paper can be accessed here:

http://www.biomedcentral.com/1471-2296/14/172/prepub

## References

[B1] Gerber Y Koton S Goldbourt U Myers V Benyamini Y Tanne D Poor neighbourhood socioeconomic status and risk of ischaemic stroke after myocardial infarction Epidemiology 2011 22 162 169 21131822 10.1097/EDE.0b013e31820463a3

[B2] Department of Health NHS Health Check: vascular risk assessment and management best practice guidance 2009 London: Department of Health

[B3] Department of Health Putting prevention first - vascular checks: risk assessment and management 2008 London: Department of Health

[B4] Stampfer MD Hu FB Manson JE Rimm EB Willet WC Primary prevention of coronary heart disease in women through diet and lifestyle New Engl J Med 2000 343 1 16 22 10882764 10.1056/NEJM200007063430103

[B5] Buck D Frosini F Clustering of unhealthy behaviours over time 2012 London: The Kings Fund

[B6] House of Commons Health Committee Health Inequalities 2009 London: The Stationery Office

[B7] Gudzune KA Clark JM Appel LJ Bennett WL Primary care providers’ communication with patients during weight counselling: a focus group study Patient Educ Couns 2012 89 1 152 157 22819710 10.1016/j.pec.2012.06.033 PMC3462265

[B8] Van der Leeuw HGA van Dijk NV Wieringa-de Warrd M Attitudes to obesity treatment in GP training practices: a focus group study Fam Pract 2011 28 4 422 429 21273284 10.1093/fampra/cmq110

[B9] Hansson LM Rasmussen F Ahlstrom GI General practitioners’ and district nurses’ conceptions of the encounter with obese patients in primary health care BMC Fam Pract 2011 12 7 21333018 10.1186/1471-2296-12-7 PMC3050702

[B10] Jansink R Braspenning J van der Weijden T Elwyn G Grol R Primary care nurses struggle with lifestyle counselling in diabetes care: a qualitative analysis BMC Fam Pract 2010 11 41 20500841 10.1186/1471-2296-11-41 PMC2889883

[B11] Arber S McKinlay J Adams A Marceau L Link C O’Donnell A How patient characteristics influence doctors’ questioning and lifestyle advice: a UK/US video experiment Brit J Gen Prac 2004 54 673 678 PMC1326068 15353053

[B12] Van Ryn M Burke J The effect of patient race and socio-economic status on physicians’ perceptions of patients Soc Sci Med 2000 50 351 357 10.1016/s0277-9536(99)00338-x 10695979

[B13] MacAuley D **The value of conducting periodic health checks** BMJ 2012 345 e7775 23169871 10.1136/bmj.e7775

[B14] McCartney M Where’s the evidence for the NHS health checks? BMJ 2013 347 f5834 24089427 10.1136/bmj.f5834

[B15] Smith S Waterall J Burden AC An evaluation of the performance of the NHS Health Check programme in identifying people at high risk of developing type 2 diabetes BMJ Open 2013 3 e002219 10.1136/bmjopen-2012-002219 PMC3612750 23468469

[B16] Cross R Accident and emergency nurses’ attitudes towards health promotion J Adv Nurs 2005 51 5 474 483 16098164 10.1111/j.1365-2648.2005.03517.x

[B17] Baker RM Economic rationality and health and lifestyle choices for people with diabetes Soc Sci Med 2006 63 2341 2353 16875768 10.1016/j.socscimed.2006.06.007

[B18] Van Excel NJA “Everyone dies so you might as well have fun!” Attitudes of Dutch youths about their health lifestyles Soc Sci Med 2006 63 10 2628 2639 16876923 10.1016/j.socscimed.2006.06.028

[B19] Watts S Stenner P Doing Q Methodological Research 2012 London: Sage

[B20] Watts S Stenner P Q methodology, quantum theory, and psychology Operant Subjectivity 2003 26 4 157 175

[B21] Stainton Rogers R Smith JA, Harre R, Van Longenhove I Q methodology Rethinking Methods in Psychology 1995 London: Sage 178 193

[B22] Bryant LD Ahmed S Ahmed M Jafri H Raashid Y ‘All is done by Allah’. Understandings of Down’s syndrome and prenatal testing in Pakistan Soc Sci Med 2011 72 8 1393 1399 21470731 10.1016/j.socscimed.2011.02.036

[B23] Murray J Craigs C Hill K Honey S House AO A systematic review of patient reported factors associated with uptake and completion of cardiovascular lifestyle behaviour change BMC Cardiovasc Disord 2012 12 120 23216627 10.1186/1471-2261-12-120 PMC3522009

[B24] Murray J Hill K Honey S Craigs C House AO Qualitative synthesis: factors affecting lifestyle change to reduce cardiovascular risk Br J Gen Pract 2012 doi:10.3399/bjgp12X649089. abridged text, in print at Br J Gen Pract 2012, **61**: 296–297 10.3399/bjgp12X649089 PMC3361119 22687232

[B25] Schmolck P Atkinson J PQMETHOD (Version 2.11) http://www.rz.unibw-muenchen.de/~p41bsmk/qmethod/

[B26] Willems S De Maesschalck S Deveugele M Derese A De Maeseneer J Socio-economic status of the patient and doctor-patient communication: does it make a difference? Patient Educ Couns 2005 56 2 139 146 15653242 10.1016/j.pec.2004.02.011

[B27] Hall JA Roter NR Katz NR Meta-analysis of correlates of provider behavior in medical encounters Med Care 1988 26 657 675 3292851 10.1097/00005650-198807000-00002

[B28] Street RL Information giving in medical consultations: the influence of patients’ communicative styles and personal characteristics Soc Sci Med 1991 32 541 548 2017721 10.1016/0277-9536(91)90288-n

[B29] Kaplan SH Gandek B Greenfield S Roger W Ware JE Patient and visit characteristics related to physicians’ participatory decision-making style Med Care 1995 33 12 1176 1187 7500658 10.1097/00005650-199512000-00002

[B30] Fiscella K Goodwin MA Strange KC Does patient education level affect office visits to family physicians J Natl Med Assoc 2002 94 3 157 165 11918385 PMC2594098

[B31] Fiscella K Epstein R So much to do, so little time Arch Intern Med 2008 168 17 1843 1852 18809810 10.1001/archinte.168.17.1843 PMC2606692

[B32] Taira DA Safran DG Seto TB Rogers WH Tarlov AR The relationship between patient income and physician discussion of health risk behaviours J Amer Med Assoc 1997 278 1412 9355999

[B33] Street RL Communicative styles and adaptations in physician-parent communication Soc Sci Med 1992 34 1155 1163 1641677 10.1016/0277-9536(92)90289-3

[B34] Johansson K Bendtsen P Akerlind I Advice to patients in Swedish primary care regarding alcohol and other lifestyle habits: how patients report the actions of GPs in relation to their own expectations and satisfaction with the consultation Eur J Public Health 2005 15 6 615 620 16093300 10.1093/eurpub/cki046

[B35] Paulhus DL Braun HI, Jackson DN, Wiley DE Socially desirable responding: the evolution of a construct The role of constructs in psychological and educational measurement 2002 New Jersey: Erlbaum 67 88

[B36] Faculty of Public Health of the Royal Colleges of Physicians of the United Kingdom Food poverty and health 2005 London: Faculty of Public health of the Royal Colleges of Physicians of the United Kingdom

[B37] Nutbeam D Health literacy as a public health goal: a challenge for contemporary health education and communication strategies into the 21st century Health Promot Int 2000 15 3 259 267

[B38] Steptoe A Doherty S Kendrick T Rink E Hilton S Attitudes to cardiovascular health promotion among GPs and practice nurses Fam Pract 1999 16 2 158 162 10381023 10.1093/fampra/16.2.158

